# Influence of Plant Growth Retardants on Quality of *Codonopsis Radix*

**DOI:** 10.3390/molecules22101655

**Published:** 2017-10-09

**Authors:** Yinyin Liao, Lanting Zeng, Pan Li, Tian Sun, Chao Wang, Fangwen Li, Yiyong Chen, Bing Du, Ziyin Yang

**Affiliations:** 1 Key Laboratory of South China Agricultural Plant Molecular Analysis and Genetic Improvement & Guangdong Provincial Key Laboratory of Applied Botany, South China Botanical Garden, Chinese Academy of Sciences, Xingke Road 723, Tianhe District, Guangzhou 510650, China; honey_yyliao@scbg.ac.cn (Y.L.); zenglanting@scbg.ac.cn (L.Z.); yychen@scbg.ac.cn (Y.C.); 2 University of Chinese Academy of Sciences, No.19A Yuquan Road, Beijing 100049, China; 3 College of Food, South China Agricultural University, Wushan Road, Tianhe District, Guangzhou 510642, China; lp19900815@126.com (P.L.); 15807662274@163.com (F.L.); 4 Tianfangjian (China) Pharmacy Company Ltd, 11 Xiancun Road, Tianhe District, Guangzhou 510623, China; Elaine.Sun@tianfangjian.com.cn; 5 Infinitus (China) Company Ltd, 11 Xiancun Road, Tianhe District, Guangzhou 510623, China; Bob.Wang@infinitus-int.com

**Keywords:** amino acid, *Codonopsis Radix*, plant growth retardant, polysaccharide, volatile

## Abstract

Plant growth retardant (PGR) refers to organics that can inhibit the cell division of plant stem tip sub-apical meristem cells or primordial meristem cell. They are widely used in the cultivation of rhizomatous functional plants; such as *Codonopsis Radix,* that is a famous Chinese traditional herb. However, it is still unclear whether PGR affects the medicinal quality of *C. Radix*. In the present study, amino acid analyses, targeted and non-targeted analyses by ultra-performance liquid chromatography combined with time-of-flight mass spectrometry (UPLC-TOF-MS) and gas chromatography-MS were used to analyze and compare the composition of untreated *C. Radix* and *C. Radix* treated with PGR. The contents of two key bioactive compounds, lobetyolin and atractylenolide III, were not affected by PGR treatment. The amounts of polysaccharides and some internal volatiles were significantly decreased by PGR treatment; while the free amino acids content was generally increased. Fifteen metabolites whose abundance were affected by PGR treatment were identified by UPLC-TOF-MS. Five of the up-regulated compounds have been reported to show immune activity, which might contribute to the healing efficacy (“buqi”) of *C. Radix*. The results of this study showed that treatment of *C. Radix* with PGR during cultivation has economic benefits and affected some main bioactive compounds in *C. Radix*.

## 1. Introduction

*Codonopsis Radix* is the dried roots of *Codonopsis pilosula*, *Codonopsis pilosulavar*, *Codonopsis modesta*, and *Codonopsis tangshen*, which are perennial herbaceous plants in the family *Campanulaceae* [1]. As one of the most famous Chinese traditional herbs, *C. Radix* has been used in clinical applications for hundreds of years. Many studies have focused on its multiple medicinal properties. It can enhance immune function, affect gastrointestinal movement, and reduce the effects of hypoxia and fatigue [2]. Furthermore, it has a sedative effect on the nervous system and can improve the function of the reproductive system [3,4]. It has also been shown to improve hematopoietic function, regulate blood pressure, strengthen heart function, and protect the circulatory system against shock [5]. With its numerous therapeutic effects and wide use in China, *C. Radix* has been studied in detail to identify the components contributing to its efficacy. So far, more than 100 pure compounds have been isolated and identified, including sterols, glycosides, alkaloids, volatile oils, triterpenoids, amino acids, phenylpropanoids, flavones, and organic acids [6]. Some of them have been shown to be major contributors to its pharmacological effects [7]. Accordingly, the types and quantities of bioactive components are critical for the efficacy of *C. Radix*.

*C. Radix* is a rhizome crop, and is often treated with plant growth retardants (PGR) to improve the biological yield. There are four different kinds of PGRs: (1) onium compounds, such as chlormequat chloride, mepiquat chloride, chlorphonium, and AMO-1618; (2) *N*-containing heterocycle, such as ancymidol, flurprimidol, tetcyclacis, paclobutrazol, uniconazole-P, and inabenfide; (3) structural analogue of 2-oxoglutaric acid, such as acylcyclohexanediones, prohexadione-Ca, trinexapac-ethyl and daminozide; and, (4) 16,17-Dihydro-GA5 and related structures [8]. Most growth retardants work by inhibiting gibberellin (GA) biosynthesis. The second type of PGR, commonly used for the cultivation of common Chinese herbs such as *Codonopsis*, *Salvia miltiorrhiza*, and Radix Ophiopogoni, was employed in the present study. While PGR certainly promotes the production of some medicinal herbs, there is some concern that it may affect the efficacy of herbal medicines, or even have toxic side-effects. Therefore, several studies have focused on the effects of PGR on the chemical composition of herb roots and their efficacy. The effect of PGR on the quality of *C. Radix* has been studied by determining the content of lobetyolin, one of the main active compounds [9]. However, the effect of PGR on the concentrations of other important compounds in *C. Radix* and on its safety are still unknown. In this study, we analyzed the contents of polysaccharides, volatile substances, and free amino acids in *C. Radix* grown with or without PGR. In addition, high-throughput screening analyses were employed to investigate whether some bioactive substances show changes in response to PGR treatment.

## 2. Results and Discussion

### 2.1. PGR Treatment of C. Radix Did Not Affect Lobetyolin and Atractylenolide III Contents

**Figure 1:**
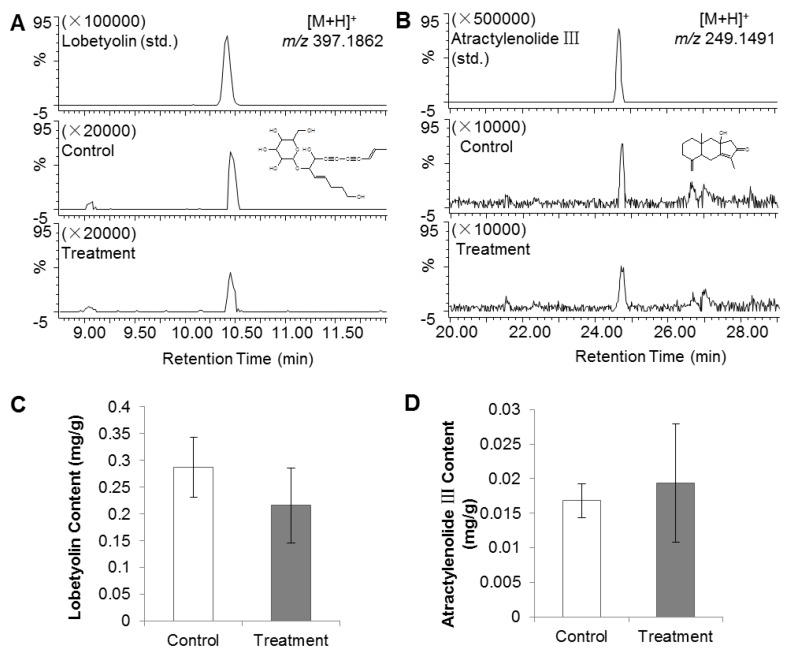
Effect of plant growth retardant (PGR) on lobetyolin and atractylenolide III contents in *C. Radix* samples. (**A**) Chromatogram comparison of lobetyolin standard, and control and treatment groups. Lobetyolin (std.), lobetyolin standard (20 ng/μL, *m*/*z* = 397.1862, RT = 10.43 min); control, untreated *C. Radix* sample; treatment, PGR-treated *C. Radix* sample. (**B**) Chromatogram comparison of atractylenolide III standard, control and treatment groups. Atractylenolide III (std.), atractylenolide III standard (10 ng/μL, *m*/*z* = 249.1491, RT = 24.68 min). (**C**) & (**D**) Lobetyolin and atractylenolide III contents in control and treatment groups (as calculated from peak areas of samples and the standard).

The contents of lobetyolin and atractylenolide III in the *C. Radix* samples were calculated by comparison with authentic standards. The average contents of lobetyolin in control and PGR-treated samples were 0.29 and 0.22 mg/g, respectively, and those of atractylenolide III were 0.017 and 0.019 mg/g, respectively. Neither compounds showed significant differences between the treated and the control groups (Figure 1).

There are two crucial active ingredients in *C. Radix*; lobetyolin and atractylenolide III. Lobetylin has been shown to have antioxidant [10], anti-inflammatory, immunity-enhancing, and stomach-protective activities, while atractylenolide III has been shown to have anti-inflammatory [11], stomach-protective [12], and anti-lung-cancer activities [13]. Since these two compounds contribute to the efficacy of *C. Radix*, many studies have analyzed changes in their abundance under various conditions or treatments, for example, sulfur fumigation, drying methods, and so on [14,15]. The results of the present study showed that these compounds showed only slight fluctuations after PGR treatment, indicating that PGR did not affect their biosynthetic pathways.

### 2.2. PGR Treatment Significantly Decreased Polysaccharides and Internal Volatiles Contents

**Figure 2:**
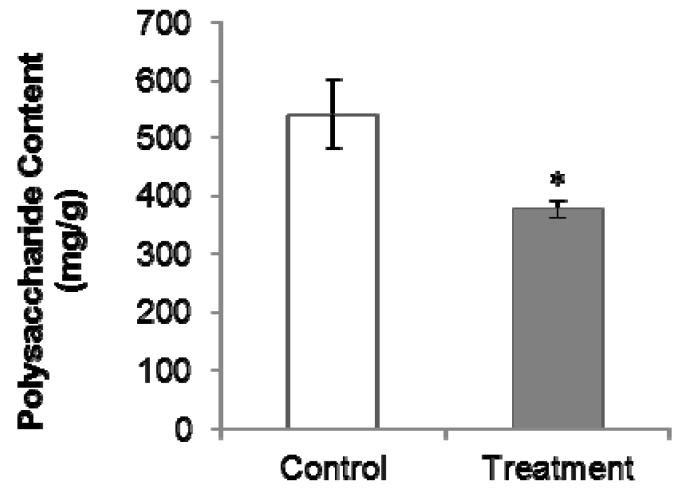
Effect of PGR on polysaccharides in *C. Radix* samples. Control, untreated *C. Radix* sample; treatment, PGR-treated *C. Radix* sample. The * indicates significant difference (*p* ≤ 0.05).

The total polysaccharides content in PGR-treated samples (376.09 mg/g) was lower than that in untreated samples (542.50 mg/g; Figure 2). Among the 39 volatile compounds detected and identified in the GC-MS analysis, 14 showed a decreased abundance in the PGR-treated samples, while the others were unaffected by PGR treatment. The 14 compounds are shown in Table 1. Diisobutyl phthalate and methyl hexadecanoate contribute to the special flavor of *C. Radix* [6].

Several studies have shown that *C. Radix* polysaccharides have bioactive properties. For example, *C. Radix* polysaccharides were shown to inhibit the growth of human gastric adenocarcinoma cells and hepatoma carcinoma cells [10], and stimulate concanavalin A or lipopolysaccharide induced lymphocyte proliferation [2]. Although there are few reports on the bioactivities of *C. Radix* volatiles, they are considered as important components [6]. Sulfur fumigation was shown to significantly alter the volatile oil and polysaccharide composition of *C. Radix* samples [6]. In our study, the contents of the main volatile oils and total polysaccharides were reduced by PGR treatment. This may be because the increase in yield under PGR treatment directed resources away from secondary metabolism. Since both growth and secondary metabolism consume nutrients and energy, an increase in yield can lead to a decrease in quality.

### 2.3. General Increase in Free Amino Acids Content by PGR Treatment

The contents of 24 common free amino acids in *C. Radix* were determined. The majority of free amino acids showed higher contents in the PGR-treated samples than in the control samples, especially asparagine, glutamic acid, α-aminoadipic acid, and ornithine (Table 2). The glycine content was significantly lower in the PGR-treated samples than in the control samples.

Free amino acids play important roles in gene expression, protein synthesis, cell signaling, metabolism, physiology, and health [16]. Many of the amino acids in *C. Radix* may be related to its effects to promote metabolism and enrich the blood and saliva. Since the main functional components of the PGR we used are mainly quaternary ammonium compounds and nitrogen-containing heterocyclic compounds, it can inhibit the biosynthesis of gibberellins [17]. This reduces the division, elongation, and growth rates of meristem cells, leading to increased tuber growth and an increased yield. These nitrogen-containing compounds can be used as substrates for amino acid synthesis. This explains why the amino acids contents generally increased after treated with PGR.

### 2.4. Application of PGR Altered the Composition of Secondary Metabolites in C. Radix

**Figure 3:**
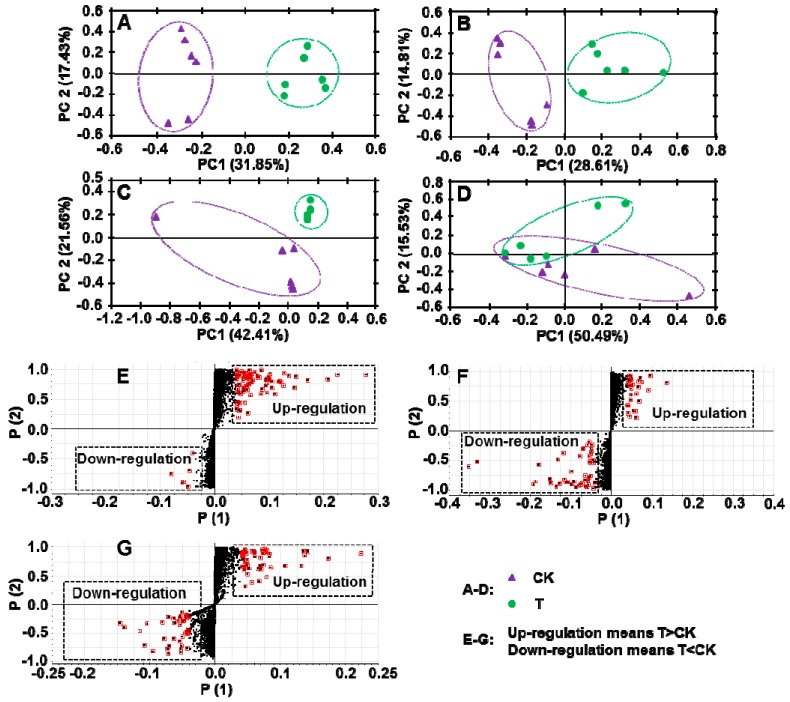
Effect of PGR on secondary metabolites in *C. Radix* samples. CK, untreated *C. Radix* sample; T, PGR-treated *C. Radix* sample. (**A**–**D**) Principal component analysis (PCA) of secondary metabolites. (**E**–**G**) S-plot analysis of secondary metabolites, red marked points were selected for further identification. (**A**, **E**) Chromatographic separation performed using ACQUITY UPLC BEH Amide column in ESI negative mode. (**B**, **F**) Chromatographic separation performed using ACQUITY UPLC BEH Amide column in ESI positive mode. (**C**, **G**) Chromatographic separation performed using ACQUITY UPLC HSS T3 C_18_ column in ESI negative mode. (**D**) Chromatographic separation performed using ACQUITY UPLC HSS T3 C_18_ column in ESI positive mode.

A principal component analysis (PCA) was used to identify differences between the PGR treated and control samples (Figure 3A–D). We detected substantial differences in all conditions, except for the C_18_ column with ESI^+^. Then, we used a S-Plot analysis to select small-molecule metabolites showing significant differences in abundance between the PGR-treated and untreated groups (Figure 3E–G). The 15 metabolites showing major differences in abundance were listed in Table 3 and Table 4. Eleven of them were up-regulated by PGR treatment (Table 3) and four of them were down-regulated by PGR treatment (Table 4).

A targeted approach, where compounds of interest are selected before further detection, has been used for a long time in the food control field. When compared with a targeted approach, a non-targeted analysis can generate data on thousands of candidates, making it possible to detect new or unexpected compounds that may improve or reduce the quality of food [18]. In the food control field, UPLC-QTOF-MS has been used to evaluate the safety of genetically modified foods, and to assess the traceability and origin of foods [19]. In this study, a non-targeted approach was used to study the effects of PGR on the quality of *C. Radix*, and many significantly affected metabolites were identified. The up-regulated compounds were mainly derivatives of fatty acids or carbohydrates, most of which are immunologically active. In a previous study, 4-mannopyranosyl-5-*O*-phosphono-ribitol had immunogenic effects and protected against the Streptococcus pneumoniae 6B capsular polysaccharide [20]. In other studies, acetylneuraminyl-galactose affected the susceptibility to infection [21], deamino neuraminyl-galactosyl-acetyl glucosamine stimulated IgG responses [22], and bougainvillein-γ-I increased resistance to radiation [23]. Aurin was reported to dramatically increase heme oxygenase-1 activity, to counteract the effects of various stressful events [24]. 9-Methylthio-2-nonanoic acid is a component of the glucosinolate biosynthesis pathway [25]. The four down-regulated compounds were mainly alkaloid derivatives whose physiological activity or toxicity is unknown. Together, the results of this study showed that PGR treatment increased the levels of several immunologically active metabolites, but did not result in the production of toxic substances.

## 3. Materials and Methods

### 3.1. Plant Materials and Treatments

Two sets of *C. Radix* samples with three repetitions (DS14A, DS14B, DS14C, and DS22A, DS22B, DS22C; finely powered) were obtained from the Infinite Pole Co., Ltd. (Guangzhou, China). The DS22 samples were treated with PGR, while the DS14 was untreated with PGR. Both of them were produced in Minxian, Gansu Province, China. The PGR, which consist of *N*-containing heterocycle, photosynthetic accelerator, rare earth fertilizer, and six trace elements, were produced by Lanzhou Yishun Industry and Trade Co., Ltd. (Lanzhou, Gansu Province, China). Its commercial name was ‘Zhuanggenling’ in Chinese (Catalog number：Agricultural fertilizer (2015) 4312). 250 mL of PGR was diluted with 15 kg water and then sprayed to 0.033 hectares of field. The PGR are generally used from late July to middle August, which is a flowering period of *C. Radix*. After treatment for about 1 month, the roots of *C. Radix* plant are harvested, dried, and crushed into powder.

### 3.2. Extraction and Analysis of Total Polysaccharides

Total polysaccharides of *C. Radix* was determined according to Reference [26] with some modifications. Finely powder samples (0.1 g) were transferred into a 50 mL triangular flask containing10 mL H_2_O and 3 mL HCl. The samples were incubated in 100 °C water for 1 h. After cooling to room temperature, the extractions were filtered, collected, and diluted with water to 250 mL. The solution (0.2 mL) was mixed with 0.8 mL H_2_O, 1 mL 5% phenol solution, and 5 mL H_2_SO_4_, and the reaction was conducted for 10 min. Absorbance of the mixture was read at 490 nm using a UV-visible spectrophotometer (UV759, Shanghai Precision Science Instrument Co., Ltd., Shanghai, China). Standard curve were prepared with solutions of 10, 20, 40, 60, 80, and 100 μg/mL glucose.

### 3.3. Extraction and Analysis of Free Amino Acids

The extraction and analysis of free amino acids was performed according to a previous study [27]. One hundred mg of sample were extracted with 1.5 mL cold methanol by vortexing for 2 min followed by ultrasonic extraction in ice water for 15 min. The extracts were mixed with 1 mL chloroform and 0.4 mL cold water for phase separation, and the resulting upper layer was dried, added with 1 mL of 5% sulfosalicylic acid, and stood for 1 h. After centrifuging at 5000× *g* for 10 min, the supernatant was filtered through 0.45 μm membrane, and subjected to an amino acid analyzer. A Sykam S433D Physiological Li C4 system (SYKAM GmbH, Eresing, Germany) equipped with quaternary pump, column oven, refrigerated auto sampler, and UV-Vis detector was used. The Physiological Li C4 system was coupled with S4300 post-column derivatization system. The experiments were performed on a high efficiency sodium cation-exchange Pickering Laboratories column (4.0 mm × 150 mm, Mountain View, CA, USA). The Sykam S433D Physiological Li C4 system was operated using a mobile phase consisting of lithium citrate pH 2.9, pH 4.2, pH 8.0, and using UV-Vis detection at 570 nm and 440 nm. The flow-rate of the mobile phase was 0.45 mL/min, and the flow-rate of the derivatizating reagent was 0.25 mL/min. The column temperature was set at 38 °C, and the post column reaction equipment was kept at 130 °C temperature. The temperature of the auto-sampler was kept at 5 °C, and the injection volume was 50 μL for both standard and samples. The content of each amino acid was calculated based on the peak area of samples and the standard.

### 3.4. Extraction and Analysis of Internal Volatiles

The extraction and analysis of internal volatiles were performed according to the previous study [28]. Two hundred mg of sample was extracted by 2 mL dichloromethane containing 4 nmol of *n*-ethyl decanoate as an internal standard in a shaker at room temperature overnight. The extraction solution was dried over anhydrous sodium sulfate and concentrated to 200 μL using nitrogen stream (MIULAB NDK200-1, MIU Instruments CO., Ltd., Hangzhou, China). Then, 1 μL of the extraction was subjected to gas chromatography-mass spectrometry (GC-MS QP2010 SE, Shimadzu Corporation, Kyoto, Japan) analysis. Samples were injected into GC injection port held at 230 °C for 1 min and all of the injections were made in splitless mode. Volatile compounds were separated on a SUPELCOWAXTM 10 column (30 m × 0.25 mm, 0.25 μm, Supelco Inc., Bellefonte, PA, USA). Helium was used as a carrier gas with a velocity 1.0 mL/min. The GC oven temperature was 60 °C for 3 min, ramp of 4 °C/min to 240 °C, and then 240 °C for 20 min. The mass spectrometry was operated with full scan mode (mass range *m/z* 40–200). The relative content of each compound from samples was calculated as GC-MS peak area ratio of analyte to ethyl decanoate (internal standard).

### 3.5. Extraction and Analysis of Characteristic Compounds and Unknown Differential Metabolites

A 10mg portion of each sample was extracted in 50 mL 70% methanol solution on ice by ultrasonic extraction. Lobetyolin and atractylenolide III were each dissolved in methanol and prepared as standard solutions. Samples were analyzed by ultra-performance liquid chromatography combined with time-of-flight mass spectrometry (UPLC-TOF-MS) using ACQUITY Class I and Xevo G2-XS instruments (Waters, Milford, MA, USA).

To comprehensively analyze compounds in the *C. Radix* samples, we used both a C_18_ column (to detect low-polarity metabolites) and an amide column (to detect high-polarity metabolites) for separations. The ESI-MS spectra were acquired in both positive and negative modes.The UPLC-MS/MS analysis using the ACQUITY UPLC HSS T3 C_18_ column (2.1 mm × 100 mm, 1.8 μm, Milford, MA, USA) was carried out using a gradient system with a flow rate of 0.3 mL/min at a column temperature of 40 °C (injection volume, 5 μL). The solvent gradient was as follows: Solvent A (Milli-Q water containing 0.1% formic acid) and solvent B (methanol); 0–30 min, 10~90% B; 30–35 min, 90% B. Individual runs were separated by an equilibration period of 5 min. The electrospray ionization (ESI)-MS/MS analysis was carried out with a Waters Q-TOF system (Milford, MA, USA). Ultra-pure nitrogen (N_2_) was used as the nebulizing and sheath gas. Product ion scanning experiments were conducted using ultra-high-purity Ar as the collision gas. The ESI parameters were as follows: Capillary voltage, 3 kV, flow rate and temperature of sheath gas, 50 L/h and 100 °C, respectively; flow rate and temperature of drying gas, 650 L/h and 350 °C, respectively; fragmentor voltage, 35~45 eV; and scan mode, 100–1000 (*m*/*z*).

The UPLC-MS/MS analysis using the ACQUITY UPLC BEH amide column (2.1 mm × 100 mm, 1.7 μm, Milford, MA, USA) was carried out with a gradient system with a flow rate of 0.3 mL/min at a column temperature of 40 °C (injection volume, 5 μL). A gradient elution of solvent A (Milli-Q water containing 10 mmol ammonium formate) and solvent B (acetonitrile containing 10 mmol ammonium formate) was applied as follows: 0–40 min, 90~60% B; 40–45 min, 60% B. Individual runs were separated by an equilibration period of 5 min. The ESI-MS/MS analysis was carried out with a Waters Q-TOF system. Ultra-pure nitrogen (N_2_) was used as the nebulizing and sheath gas. Product ion scanning experiments were conducted using ultra-high-purity Ar as the collision gas. The ESI parameters were as follows: capillary voltage, 3 kV; flow rate and temperature of sheath gas, 50 L/h and 100 °C, respectively; flow rate and temperature of drying gas, 650 L/h and 350 °C, respectively; fragmentor voltage, 25~35 eV; and scan mode, 100–1000 (*m*/*z*).

Firstly, a PCA was used to identify the differences between the PGR treated and control samples. Then, we used S-Plot analysis to select small molecule metabolites showing significant differences in abundance between the PGR treated and untreated groups. The candidates were identified based on comparison, with 595 commercial databases, mass spectra fragments, and published papers.

### 3.6. Data Analysis

Statistical analysis was performed using the SPSS Ver. 19.0 software (SPSS Inc., Chicago, IL, USA). The paired and independent samples *t*-tests were used to determine differences between two groups. A probability level of 5% (*p* ≤ 0.05) was considered significant.

## 4. Conclusion

**Figure 4:**
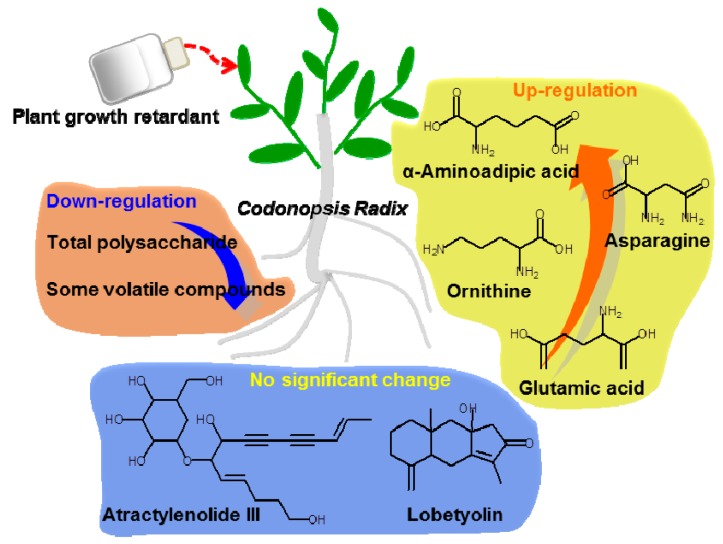
Summary on effect of PGR on *C. Radix* samples.

The use of PGR can increase the biomass of *C. Radix* and change the distribution of its metabolites. In the present study, the effect of PGR on the active substances and chemical composition of *C. Radix* was comprehensively investigated (Figure 4). Application of PGR did not affect the contents of lobetyolin and atractylenolide III. The polysaccharides content and internal volatiles content were significantly lower in PGR-treated samples than in control samples. The free amino acid content was generally increased in PGR-treated samples. In a non-targeted analysis, 15 metabolites were identified to be affected by PGR treatment. Five of the up-regulated compounds have been reported to show immune activity. No toxic substances were detected. Together, these results indicate that application of PGR during the cultivation of *C. Radix* is of economic benefit, and affects some of the main bioactive components contributing to its overall pharmacological effects.

## Figures and Tables

**Table 1:** Effect of PGR on internal volatiles in *C. Radix* samples.

Internal Volatile Metabolite	Treatment	Control	T/CK
Dodecane	0.05 ± 0.01	0.09 ± 0.01	0.56 *
Hexadecane	0.13 ± 0.01	0.23 ± 0.02	0.57 *
2-Furanmethanol	0.06 ± 0.01	0.13 ± 0.01	0.46 *
Phytane	0.10 ± 0.01	0.17 ± 0.03	0.59 *
Octacosane	0.05 ± 0.01	0.10 ± 0.01	0.50 *
Dihydroanethole	0.27 ± 0.10	1.42 ± 0.28	0.19 *
Oxalic acid, dodecyl 2-methylphenyl ester	0.05 ± 0.01	0.11 ± 0.01	0.46 *
Ethylhexyl benzoate	0.04 ± 0.01	0.07 ± 0.00	0.57 *
Methyl hexadecanoate	0.16 ± 0.01	0.24 ± 0.03	0.67 *
Diethyl Phthalate	0.10 ± 0.01	0.14 ± 0.01	0.71 *
n-Heptadecylcyclohexane	0.03 ± 0.00	0.06 ± 0.01	0.50 *
Methyl octadeca-9,12-dienoate	0.11 ± 0.01	0.21 ± 0.04	0.52 *
Diisobutyl phthalate	0.72 ± 0.08	1.10 ± 0.10	0.66 *
Phthalic acid, decyl 2,7-dimethyloct-7-en-5-yn-4-yl ester	0.54 ± 0.07	0.79 ± 0.03	0.69 *

The relative content of each compound from samples was calculated as GC-MS peak area ratio of analyte to *n*-ethyl decanoate (internal standard). Data are expressed as mean ± SD (*n* = 3). Control, untreated *C. Radix* sample; treatment, PGR-treated *C. Radix* sample. T/CK represents ratio of internal volatile content in PGR treatment to that in control. The * indicates significant difference (*p* ≤ 0.05).

**Table 2:** Effect of PGR on free amino acids in *C. Radix* samples.

Free Amino Acid	Treatment (μg/g)	Control (μg/g)	T/CK
Glutamic acid	3234.18 ± 811.83	465.59 ± 174.63	6.95 *
Asparagine	172.88 ± 44.88	34.36 ± 15.40	5.03 *
α-Aminoadipic Acid	3270.40 ± 898.05	659.44 ± 244.17	4.96 *
Ornithine	13.08 ± 1.59	6.41 ± 2.04	2.04 *
Taurine	121.03 ± 85.58	7.56 ± 5.38	16.01
Phosphatidylserine	32.91 ± 16.27	10.05 ± 1.71	3.27
Carbamide	17619.48 ± 7948.53	9652.14 ± 3208.69	1.83
Leucine	22.65 ± 7.09	12.91 ± 4.08	1.75
Arginine	5431.27 ± 1205.33	3164.94 ± 1227.21	1.72
Serine	47.96 ± 11.59	32.21 ± 12.45	1.49
Citrulline	214.58 ± 74.13	149.97 ± 60.34	1.43
β-Aminoisobutyric acid	1.72 ± 0.42	1.28 ± 0.58	1.34
Valine	12.34 ± 2.41	9.42 ± 3.65	1.31
Isoleucine	61.95 ± 20.73	51.04 ± 16.87	1.21
Lysine	50.74 ± 9.78	44.94 ± 18.04	1.13
β-Alanine	4.93 ± 2.66	4.47 ± 2.49	1.10
Phenylalanine	18.95 ± 4.69	17.45 ± 5.79	1.09
Alanine	270.21 ± 102.56	291.28 ± 111.14	0.93
Glycine	0.00 ± 0.00	3.20 ± 1.10	0.00 *
Tyrosine	8.98 ± 2.76	11.19 ± 4.72	0.80
Histidine	2448.93 ± 786.52	2454.37 ± 360.11	1.00
γ-Aminobutyric acid	27.76 ± 8.04	44.82 ± 10.31	0.62
Tryptophan	21.32 ± 8.14	37.35 ± 17.81	0.57
Threonine	9.96 ± 5.08	45.86 ± 53.54	0.22

Content of each amino acid was calculated based on the peak area of samples and the standard. Data are expressed as mean ± SD (*n* = 3). Control, untreated *C. Radix* sample; treatment, PGR-treated *C. Radix* sample. T/CK represents ratio of content in PGR treatment to that in control. The * indicates significant difference (*p* ≤ 0.05).

**Table 3:** Major secondary metabolites up-regulated by PGR.

No.	Identification and Tentative Identification	Molecular Formula	RT (min)	*m/z*	T/CK	Mode
1	Arginine	C_6_H_14_N_4_O_2_	0.70	173.1045[M − H]^−^	1.57 *	C_18_, ES^−^
2	Verbascose	C_30_H_52_O_26_	0.75	827.2670[M − H]^−^	1.71 **	C_18_, ES^−^
3	(4-Aminomethyl-1,2,3-triazol-1-acetylamino)-5-hydroxybenzoic acid	C_12_H_13_N_5_O_4_	0.82	290.0883 [M − H]^−^	2.23 **	C_18_, ES^−^
4	4-Mannopyranosyl-5-*O*-phosphono-ribitol	C_11_H_23_O_12_P	0.82	377.0859 [M − H]^−^	1.62 *	C_18_, ES^−^
5	Acetylneuraminyl-galactose	C_17_H_29_NO_14_	0.82	470.1523 [M − H]^−^	2.67 **	C_18_, ES^−^
6	Deamino neuraminyl-galactosyl-acetyl glucosamine	C_23_H_39_NO_19_	0.82	632.2041 [M − H]^−^	3.19 **	C_18_, ES^−^
7	Glycoloyloxy-acetic acid	C_4_H_6_O_5_	0.87	133.0144 [M − H]^−^	1.51 **	C_18_, ES^−^
8	Bougainvillein-γ-I	C_30_H_36_N_2_O_18_	11.68	729.2257[M − NH_4_]^−^	1.25	Amide, ES^−^
9	Aurin	C_19_H_14_O_3_	20.27	307.1207[M+NH_4_]^−^	1.72 **	Amide, ES^−^
10	9-Methylthio-2-nonanoic acid	C_10_H_18_O_3_S	20.32	217.0906 [M − H]^−^	1.38 **	Amide, ES^−^
11	5,6,7,8-Tetrahydromethanopterin	C_30_H_45_N_6_O_16_P	7.20	794.3002[M+NH_4_]	6.29 **	Amide, ES^+^

CK, untreated *C. Radix* sample; T, PGR-treated *C. Radix* sample. T/CK, ratio of metabolite’s peak area in PGR-treated group to that in control group; The * and ** indicate significant differences (*p* ≤ 0.05 and *p* ≤ 0.01, respectively).

**Table 4:** Major secondary metabolites down-regulated by PGR.

No.	Identification and Tentative Identification	Molecular Formula	RT (min)	*m/z*	T/CK	Mode
1	3-Hexadecanoylamino-2-hydroxy-4-methylpentyl-phosphonic acid	C_22_H_46_NO_5_P	1.05	453.3469[M + NH_4_]	0.02 **	Amide, ES^+^
2	4,4′-Dioxo-carotene-3,3′-diyl didecanoate	C_60_H_88_O_6_	1.05	905.6666[M + H]^+^	0.01 **	Amide, ES^+^
3	1-Dimethylylidene-4-dihydrazinecarboximidamide-2,3-dimethoxybenzene	C_12_H_18_N_8_O_2_	4.18	324.1880[M + NH_4_]	0.08 **	Amide, ES^+^
4	Phenylalanyl-glycyl-histidine	C_17_H_21_N_5_O_4_	1.74	358.1504 [M − H]^−^	0.27 **	C_18_, ES^−^

CK, untreated *C. Radix* sample; T, PGR-treated *C. Radix* sample. T/CK, ratio of metabolite’s peak area in PGR-treated group to that in control group; The * and ** indicate significant differences (*p* ≤ 0.05 and *p* ≤ 0.01, respectively).

## References

[1] Chinese Pharmacopoeia Commission (2010). Pharmacopoeia of the People’s Republic of China (in Chinese).

[2] Sun Y.X., Liu J.C. (2008). Structural characterization of a water-soluble polysaccharide from the roots of *Codonopsis pilosula* and its immunity activity. Int. J. Biol. Macromol..

[3] Hu H.G., Sun W.J., Xiao X., Tang X.J., Hu Q.L., Xu S.F. (2014). Water extract from *Codonopsis thalictrifolia* wall affects the reproductive system of male infant rats. Nat. J. Androl..

[4] Weon J.B., Yun B.R., Lee J., Eom M.R., Ko H.J., Lee H.Y., Park D.S., Chung H.C., Chung J.Y., Ma C.J. (2014). Neuroprotective effect of steamed and fermented *Codonopsis Lanceolata*. Biomol. Ther..

[5] Qin L.M., Yan Y.F., Wang Z.C. (1994). Experimental study on the cardiotonic action of extract from *Codonopsis pilosula* (Franch.) Nannf (in Chinese). China J. Chin. Mater. Medica.

[6] He J.Y., Ma N., Zhu S., Komatsu K., Li Z.Y., Fu W.M. (2015). The genus *Codonopsis* (Campanulaceae): A review of phytochemistry, bioactivity and quality control. J. Nat. Med..

[7] Kim E.Y., Kim J.A., Jeon H.J., Kim S., Kim Y.H., Kim Y.H., Kim H.Y., Whang W.K. (2014). Chemical fingerprinting of *Codonopsis pilosula* and simultaneous analysis of its major components by HPLC-UV. Arch. Pharm. Res..

[8] Rademacher W. (2000). Growth retardants: Effects on gibberellin biosynthesis and other metabolic pathways. Annu. Rev. Plant Physiol. Plant Mol. Biol..

[9] Chen Y.W., Ding Y.H., Li C.Y., Chen F.Q., Yang S.T., Chen J., Li S., Gao X.Y. (2011). Study on effects of Dangshen Zhuanggenling on quality of *Codondpisis Pilosula* (Franch.) Nannf (in Chinese). Chin J. Pharm. Anal..

[10] Dumlu M.U., Gurkan E., Tuzlaci E. (2008). Chemical composition and antioxidant activity of *Campanula alliariifolia*. Nat. Prod. Res..

[11] Li C.Q., He L.C., Jin J.Q. (2007). Atractylenolide I and atractylenolide III inhibit lipopolysaccharide-induced TNF-α and NO production in macrophages. Phytother. Res..

[12] Wang K.T., Chen L.G., Wu C.H., Chang C.C., Wang C.C. (2010). Gastroprotective activity of atractylenolide III from *Atractylodes Ovata* on ethanol-induced gastric ulcer in vitro and in vivo. J. Pharm. Pharmacol..

[13] Kang T.H., Bang J.Y., Kim M.H., Kang I.C., Kim H.M., Jeong H.J. (2011). Atractylenolide III, a sesquiterpenoid, induces apoptosis in human lung carcinoma A549 cells via mitochondria-mediated death pathway. Food Chem. Toxicol..

[14] Peng R., Ma P., Sun N.N., Li L.Y. (2010). Effect of fertilization on the yield and quality of *Codonopsis* Tangshen Oliv (in Chinese). World Sci. Technol..

[15] Yang F.R., Li Z.M., Gao J.P. (2011). Separation and structural characterization and anti-tumor effect in vitro of polysaccharides from *Radix Codonopsis* (in Chinese). Lishizhen Med. Mater. Medica Res..

[16] Kong X.F., Wu G., Yin Y.L. (2011). Roles of phytochemicals in amino acid nutrition. Front. Biosci..

[17] Li X.E., Zhang X.Y. (2014). Influence of plant growth regulater on yield and quality of *Salvia Miltiorrhiza* (in Chinese). China J. Chin. Mater. Medica.

[18] Tengstrand E., Rosén J., Hellenäs K.E., Åberg K.M. (2013). A concept study on non-targeted screening for chemical contaminants in food using liquid chromatography-mass spectrometry in combination with a metabolomics approach. Anal. Bioanal. Chem..

[19] Herreroyr M., Simó C., García-Cañas V., Ibáñez E., Cifuentes A. (2012). Foodomics: MS-based strategies in modern food science and nutrition. Mass Spectrom. Rev..

[20] Jansen W.T., Hogenboom S., Thijssen M.J., Kamerling J.P., Vliegenthart J.F., Verhoef J. (2001). Synthetic 6B di-, tri-, and tetrasaccharide-protein conjugates contain pneumococcal type 6A and 6B common and 6B-specific epitopes that elicit protective antibodies in mice. Infect. Immun..

[21] Thorpe S.J., Feizi T. (1984). Species differences in the expression of carbohydrate differentiation antigens on mammalian blood cells revealed by immunofluorescence with monoclonal antibodies. Biosci. Rep..

[22] Von G.S., Smith D.F., Cummings R.D., Riedel S., Miescher S., Schaub A. (2009). Intravenous immunoglobulin contains a broad repertoire of anticarbohydrate antibodies that is not restricted to the IgG 2 subclass. J. Allergy Clin. Immun..

[23] Truong N.M., Dang Q.T. (2016). Application of hydrolytic enzymes for improvement of red dragon fruit juice processing. Asian Pac. J. Sustain. Agr. Food Energ..

[24] Foresti R., Hoque M., Monti D., Green C.J., Motterlini R. (2005). Differential activation of heme oxygenase-1 by chalcones and rosolic acid in endothelial cells. J. Pharmacol. Exp. Ther..

[25] Mikkelsen M.D., Halkier B.A. (2003). Metabolic engineering of valine and isoleucine-derived glucosinolates in Arabidopsis expressing CYP79D2 from cassava. Plant Physiol..

[26] Zheng T., Liu C.C., Yang J.Y., Liu Q.G., Li J.L. (2013). Hijiki Seaweed (*Hizikia fusiformis*): Nutritional value, safety concern and arsenic removal method. Adv. Mater. Res..

[27] Chen Y.Y., Fu X.M., Mei X., Zhou Y., Cheng S.H., Zeng L.T., Dong F., Yang Z.Y. (2017). Proteolysis of chloroplast proteins is responsible for accumulation of free amino acids in dark-treated tea (*Camellia sinensis*) leaves. J. Proteom..

[28] Fu X.M., Cheng S.H., Zhang Y.Q., Du B., Feng C., Zhou Y., Mei X., Jiang Y.M., Duan X.W., Yang Z.Y. (2017). Differential responses of four biosynthetic pathways of aroma compounds in postharvest strawberry (*Fragaria × ananassa* Duch.) under interaction of light and temperature. Food Chem..

